# Correction: Explaining public understanding of the concepts of climate change, nutrition, poverty and effective medical drugs: An international experimental survey

**DOI:** 10.1371/journal.pone.0237445

**Published:** 2020-08-05

**Authors:** The PLOS ONE Staff

## Notice of republication

This article was republished on July 10, 2020, to correct an error in the placement of the tables and figures: they were erroneously placed ahead of its first in-text citation during the typesetting process. The publisher apologizes for the error. Please download this article again to view the correct version. The originally published, uncorrected article and the republished, corrected article are provided here for reference.

## Supporting information

S1 File Originally published, uncorrected article. (PDF)

S2 File Republished, corrected article. (PDF)
