# Supplementary material for: Explaining public understanding of the concepts of climate change, nutrition, poverty and effective medical drugs: An international experimental survey
Source: PLoS One. 2020 Jun 10;15(6):e0234036. doi: 10.1371/journal.pone.0234036 (PMC7286496; doi:10.1371/journal.pone.0234036)
Supplement: S1 File — (DOCX) [file pone.0234036.s001.docx]

**Appendix: Survey questionnaire**

**Values in science – experimental group 1**

Intro Values in Science Survey

  The objective of this study is to gain better insight into how people understand four scientific concepts involved in four global issues: *healthy nutrition*, *climate change*, *poverty*, and *effective medications*.

The study consists of a survey in three parts. The first part includes 40 short questions, where you'll have to think about the public understanding of nutrition, climate change, poverty and medicine. The second part includes 4 questions concerning ways of improving public understanding of science. The final part includes 15 questions about your own demographic background. Completing the survey will take approximately 15-20 minutes.
   Participation in this research study is completely voluntary. The study material is not harmful in any respect. Withdrawal from participation is possible at any time. To this end, simply close the window of your internet browser. To receive your payment at the end of the survey, you will have to answer all questions, and you should enter the correct survey code at the end of the survey.

Confidentiality. Data will be collected, stored, and processed in anonymised form, in compliance with the data protection laws of Tilburg University, The Netherlands. Data will be exclusively evaluated on group levels.

If you agree to participate in the study, then please click the agree option below.  If you do not agree to participate in the study, then please close your browser window to exit from the study.

- I have read the informed consent statements above and I agree to participate in this study.

Nut1.1 Please indicate how strongly you agree or disagree with the following statements.

The general public can explain what it is that makes nutrition *healthy*. *(‘The general public’ refers to ordinary people across the world who do not have any special type of expertise on this issue.)*

- Strongly agree
- Somewhat agree
- Neither agree nor disagree
- Somewhat disagree
- Strongly disagree

Clim1.1 The general public can explain what causes *climate change*.

- Strongly agree
- Somewhat agree
- Neither agree nor disagree
- Somewhat disagree
- Strongly disagree

Pov1.1 The general public can explain what causes *poverty*.

- Strongly agree
- Somewhat agree
- Neither agree nor disagree
- Somewhat disagree
- Strongly disagree

Med1.1 The general public can explain what it is that makes a medical drug *effective*.

- Strongly agree
- Somewhat agree
- Neither agree nor disagree
- Somewhat disagree
- Strongly disagree

Nut1.2 What do you think is the level of disagreement within the scientific community about how *healthy* nutrition should be defined?

- 1. Strong agreement
- 2
- 3
- 4
- 5. Strong disagreement

*Skip To: Nut4.1 If What do you think is the level of disagreement within the scientific community about how healthy... = 1. Strong agreement*

Nut3.1 What reasons do you believe are the causes of the lack of agreement?

 Indicate how much each of the following factors contributes to the disagreement:

|  | 1 - strong contribution | 2 | 3 | 4 | 5 - no contribution |
| --- | --- | --- | --- | --- | --- |
| Complexity of the topic |  |  |  |  |  |
| Contradicting scientific studies on the topic |  |  |  |  |  |
| Lack of objective (accurate) facts about the topic |  |  |  |  |  |
| Biased scientific studies on the topic (over- or under-estimated results) |  |  |  |  |  |
| Selective reporting of results (researchers only reporting positive results or particular results in their published studies) |  |  |  |  |  |
| Fraud (fabricated or falsified results) |  |  |  |  |  |
| Conflict of interest |  |  |  |  |  |

Nut4.1 I believe it is the responsibility of the government to ensure that the general public understands what *healthy* nutrition is.

- Strongly agree
- Somewhat agree
- Neither agree nor disagree
- Somewhat disagree
- Strongly disagree

Nut5.1 Which one of these possible definitions of *healthy* nutrition do you believe is the most widely accepted within the scientific community?

- Healthy nutrition is the result of eating any food in whatever amount that makes one feel good.
- Healthy nutrition is the result of eating a variety of foods, plenty of vegetables and fruit, moderate amounts of fats and oils and less salt and sugars.
- Healthy nutrition is the result of eating only vegetables, fruit, legumes, mushrooms and nuts, and avoiding all animal foods including milk and honey.

Nut6.1  Which one of these possible explanations of what makes nutrition *healthy* do you believe is the most widely accepted within the scientific community?

- Healthy nutrition is how we defend ourselves against disease and aging. Poor nutrition can lead to lower bodily functions, and reduced levels of physical output and effectiveness.
- Healthy nutrition is how we acquire energy and the building blocks for our bodily system. Poor nutrition can lead to inadequate energy, and low mental functioning and efficiency.
- Healthy nutrition is how we attain good health through an adequate, well balanced diet. Poor nutrition can lead to reduced immunity, increased susceptibility to disease, impaired physical and mental development, and reduced productivity.

Nut7.1 Think again about your knowledge of how nutrition works.
 To what extent do you agree that you can explain what *healthy* nutrition is?

- Strongly agree
- Somewhat agree
- Neither agree nor disagree
- Somewhat disagree
- Strongly disagree

Nut8.1 To what extent do you agree the scientific community can provide a definition of *healthy* nutrition without making value-judgements (that is, without appealing to somebody’s opinions about what types of things are good or bad for someone)?

- Strongly agree
- Somewhat agree
- Neither agree nor disagree
- Somewhat disagree
- Strongly disagree

Nut9.1 To what extent do you agree that the scientific community can provide an objective (accurate) definition of *healthy* nutrition?

- Strongly agree
- Somewhat agree
- Neither agree nor disagree
- Somewhat disagree
- Strongly disagree

Nut10.1 Has your knowledge about *healthy* nutrition improved in the past 10 years?

- Yes
- No

*Skip To: Nut11.1 If Has your knowledge about healthy nutrition improved in the past 10 years? = No*

Nut10a.1 Has your nutrition changed in the past 10 years, based on your new knowledge?

- Strongly agree
- Somewhat agree
- Neither agree nor disagree
- Somewhat disagree
- Strongly disagree

Nut11.1 Would you be in favour of your government levying a tax for everyone that increases by 10% the price of all unhealthy foods, and these additional revenues are invested in public information campaigns about the effects of unhealthy foods?

- Strongly in favour
- Somewhat in favour
- Neither in favour nor against
- Somewhat against
- Strongly against

Clim2.1 What do you think is the level of disagreement within the scientific community about how *climate change* should be defined?

- 1. Strong agreement
- 2
- 3
- 4
- 5. Strong disagreement

*Skip To: Clim4.1 If What do you think is the level of disagreement within the scientific community about how climate... = 1. Strong agreement*

Clim3.1 What reasons do you believe are the causes of the lack of agreement?
 Indicate how much each of the following factors contributes to the disagreement:

|  | 1 - strong contribution | 2 | 3 | 4 | 5 - no contribution |
| --- | --- | --- | --- | --- | --- |
| Complexity of the topic |  |  |  |  |  |
| Contradicting scientific studies on the topic |  |  |  |  |  |
| Lack of objective (accurate) facts about the topic |  |  |  |  |  |
| Biased scientific studies on the topic (over- or under-estimated results) |  |  |  |  |  |
| Selective reporting of results (researchers only reporting positive results or particular results in their published studies) |  |  |  |  |  |
| Fraud (fabricated or falsified results) |  |  |  |  |  |
| Conflict of interest |  |  |  |  |  |

Clim4.1 I believe it is the responsibility of the government to ensure that the general public understands what *climate change* is.

- Strongly agree
- Somewhat agree
- Neither agree nor disagree
- Somewhat disagree
- Strongly disagree

Clim5.1 Which one of these possible definitions of *climate change* do you believe is the most widely accepted within the scientific community?

- A change of the climate that takes place at regular intervals during each year, decade, or century, and is characterised by changes in amount of daylight, rain, and temperature.
- A change of climate which is attributed directly or indirectly to human activity that alters the composition of the global atmosphere and which is in addition to natural climate variability observed over comparable time periods.
- A change of the climate that takes place at irregular intervals during each year, and is characterised by changes in the extension of deserts, forests, and glaciers.

Clim6.1 Which one of these possible explanations of how *climate change* takes place do you believe is the most widely accepted within the scientific community?

- Climate change takes place because of natural processes such as modulations of the solar cycles, volcanic eruptions, and also because of persistent anthropogenic changes in the composition of the atmosphere or in land use.
- Climate change takes place because of natural processes such as seasonal patterns, oceans tides, and also because of persistent natural changes in the position of the North Pole or the distance of the Sun to the Earth.
- Climate change takes place because of natural processes such as modulations of atmospheric pressure, earthquakes, and also because of persistent anthropogenic changes in the extension of deserts or in pesticide use.

Clim7.1 Think again about your knowledge of how climate change takes place.

 To what extent do you agree that you can explain what *climate change* is?

- Strongly agree
- Somewhat agree
- Neither agree nor disagree
- Somewhat disagree
- Strongly disagree

Clim8.1 To what extent do you agree the scientific community can provide a definition of *climate change* without making value-judgements (that is, without appealing to somebody’s opinions about what types of things are good or bad for someone)?

- Strongly agree
- Somewhat agree
- Neither agree nor disagree
- Somewhat disagree
- Strongly disagree

Clim9.1 To what extent do you agree that the scientific community can provide an objective (accurate) definition of *climate change*?

- Strongly agree
- Somewhat agree
- Neither agree nor disagree
- Somewhat disagree
- Strongly disagree

Clim10.1 Has your knowledge about *climate change* improved in the past 10 years?

- Yes
- No

*Skip To: Clim11.1 If Has your knowledge about climate change improved in the past 10 years? = No*

Clim10a.1 Has your behavior changed in relation to climate change in the past 10 years, based on your new knowledge?

- Strongly agree
- Somewhat agree
- Neither agree nor disagree
- Somewhat disagree
- Strongly disagree

Clim11.1 Would you be in favour of your government levying a tax for everyone that increases by 10% the price of all goods you consume that produce high levels of CO2 emissions (airplane tickets, conventional electricity at home, purchasing meat etc), and these additional revenues are invested in CO2 reducing measures?

- Strongly in favour
- Somewhat in favour
- Neither in favour nor against
- Somewhat against
- Strongly against

Pov2.1 What do you think is the level of disagreement within the scientific community about how *poverty* should be defined?

- 1. Strong agreement
- 2
- 3
- 4
- 5. Strong disagreement

*Skip To: Pov4.1 If What do you think is the level of disagreement within the scientific community about how poverty... = 1. Strong agreement*

Pov3.1 What reasons do you believe are the causes of the lack of agreement?

 Indicate how much each of the following factors contributes to the disagreement:

|  | 1 - strong contribution | 2 | 3 | 4 | 5 - no contribution |
| --- | --- | --- | --- | --- | --- |
| Complexity of the topic |  |  |  |  |  |
| Contradicting scientific studies on the topic |  |  |  |  |  |
| Lack of objective (accurate) facts about the topic |  |  |  |  |  |
| Biased scientific studies on the topic (over- or under-estimated results) |  |  |  |  |  |
| Selective reporting of results (researchers only reporting positive results or particular results in their published studies) |  |  |  |  |  |
| Fraud (fabricated or falsified results) |  |  |  |  |  |
| Conflict of interest |  |  |  |  |  |

Pov4.1 I believe it is the responsibility of the government to ensure that the general public understands what *poverty* is.

- Strongly agree
- Somewhat agree
- Neither agree nor disagree
- Somewhat disagree
- Strongly disagree

Pov5.1 Which one of these possible definitions of *poverty* do you believe is the most widely accepted within the scientific community?

- Poverty is the inability to provide oneself with shelter, clothes, and food, which would allow for survival, and reaching a standard that permits to eat good enough food and attain good enough health.
- Poverty is the inability to attain a minimal standard of living, encompassing not only consumption of food, clothing, and shelter, but also access to education, health services and clean water.
- Poverty is the inability to provide oneself with the means to choose the life one would like to lead given a lack of opportunities to good food and good health.

Pov6.1 Which one of these possible explanations of *poverty* do you believe is the most widely accepted within the scientific community?

- Poverty is caused by individual laziness, bad choices, incompetence, or inherent disabilities preventing certain individuals from having an education, a job, a house, or a large social network of friends.
- Poverty is caused by societal barriers preventing certain individuals from participating in institutions such as education, healthcare, housing, the job market and political representation.
- Poverty is caused by a lack of income and assets to attain basic necessities (food, shelter, clothing, and acceptable levels of health and education), a sense of voicelessness and powerlessness in the institutions of state and society, and vulnerability to adverse shocks, linked to an inability to cope with them.

Pov7.1 Think again about your knowledge of how poverty comes about.

 To what extent do you agree that you can explain what *poverty* is?

- Strongly agree
- Somewhat agree
- Neither agree nor disagree
- Somewhat disagree
- Strongly disagree

Pov8.1 To what extent do you agree the scientific community can provide a definition of *poverty* without making value-judgements (that is, without appealing to somebody’s opinions about what types of things are good or bad for someone)?

- Strongly agree
- Somewhat agree
- Neither agree nor disagree
- Somewhat disagree
- Strongly disagree

Pov9.1 To what extent do you agree that the scientific community can provide an objective (accurate) definition of *poverty*?

- Strongly agree
- Somewhat agree
- Neither agree nor disagree
- Somewhat disagree
- Strongly disagree

Pov10.1 Has your knowledge about *poverty* improved in the past 10 years?

- Yes
- No

*Skip To: Pov11.1 If Has your knowledge about poverty improved in the past 10 years? = No*

Pov10a.1 Has your behavior changed in relation to poverty in the past 10 years, based in your new knowledge?

- Strongly agree
- Somewhat agree
- Neither agree nor disagree
- Somewhat disagree
- Strongly disagree

Pov11.1  Would you be in favour of your government levying a tax for everyone that increases by 3% the price on all goods (so that if you for example now spend $100 on some item, you will then spend $103 on the same item), and these additional revenues are invested in poverty reduction programmes?

- Strongly in favour
- Somewhat in favour
- Neither in favour nor against
- Somewhat against
- Strongly against

Med2.1 What do you think is the level of disagreement within the scientific community about how the effectiveness of medical drugs should be defined?

- 1. Strong agreement
- 2
- 3
- 4
- 5. Strong disagreement

*Skip To: Med4.1 If What do you think is the level of disagreement within the scientific community about how the effe... = 1. Strong agreement*

Med3.1 What reasons do you believe are the causes of the lack of agreement?

|  | 1 - strong contribution | 2 | 3 | 4 | 5 - no contribution |
| --- | --- | --- | --- | --- | --- |
| Complexity of the topic |  |  |  |  |  |
| Contradicting scientific studies on the topic |  |  |  |  |  |
| Lack of objective (accurate) facts about the topic |  |  |  |  |  |
| Biased scientific studies on the topic (over- or under-estimated results) |  |  |  |  |  |
| Selective reporting of results (researchers only reporting positive results or particular results in their published studies) |  |  |  |  |  |
| Fraud (fabricated or falsified results) |  |  |  |  |  |
| Conflict of interest |  |  |  |  |  |

Med4.1 I believe it is the responsibility of the government to ensure that the general public understands what counts as an *effective* drug.

- Strongly agree
- Somewhat agree
- Neither agree nor disagree
- Somewhat disagree
- Strongly disagree

Med5.1 Which one of these possible definitions of the effectiveness of a medical drug do you believe is the most widely accepted within the scientific community?

- Effectiveness is the extent to which a drug increases one’s life expectancy.
- Effectiveness is the extent to which a drug does what it is intended to do, when it’s taken in the appropriate circumstances.
- Effectiveness is the extent to which a drug lowers one’s suffering if recipients adhere to the proper dosage.

Med6.1 Which one of these possible explanations of the *effectiveness* of a medical drug do you believe is the most widely accepted within the scientific community?

- It is the capacity for a drug to illustrate positive returns on people’s health which is determined by conducting rigorous qualitative studies.
- It is the capacity of medical practitioners to collect and assess the effects of drugs taken by their patients and establish which drugs are most beneficial for their patients over time. This is conducted through rigorous protocols.
- It is the capacity for a beneficial effect of a given drug intervention. Establishment of the effectiveness of an intervention is often done relative to other available interventions, with which it is compared. It refers to whether a drug demonstrates a health benefit over a placebo or other intervention when tested such as in controlled clinical trials.

Med7.1 Think again about your knowledge of how the effectiveness of medical drugs.

 To what extent do you agree that you can explain what it is that makes a drug *effective*?

- Strongly agree
- Somewhat agree
- Neither agree nor disagree
- Somewhat disagree
- Strongly disagree

Med8.1 To what extent do you agree the scientific community can provide a definition of the *effectiveness* of medical drugs without making value-judgements (that is, without appealing to somebody’s opinions about what types of things are good or bad for someone)?

- Strongly agree
- Somewhat agree
- Neither agree nor disagree
- Somewhat disagree
- Strongly disagree

Med9.1 To what extent do you agree that the scientific community can provide an objective (accurate) definition of the *effectiveness* of medical drugs?

- Strongly agree
- Somewhat agree
- Neither agree nor disagree
- Somewhat disagree
- Strongly disagree

Med10.1 Has your knowledge about what it is that makes a drug *effective* improved in the past 10 years?

- Yes
- No

*Skip To: Med11.1 If Has your knowledge about what it is that makes a drug effective improved in the past 10 years? = No*

Med10a.1 Has your behavior changed in relation to medical drugs in the past 10 years, based on your new knowledge?

- Strongly agree
- Somewhat agree
- Neither agree nor disagree
- Somewhat disagree
- Strongly disagree

Med11.1 Would you be in favour of your government levying a tax for everyone that increases by 10% the price of each medical drug, and these additional revenues are invested in public campaigns to better inform citizens about medical drugs and what makes them effective?

- Strongly in favour
- Somewhat in favour
- Neither in favour nor against
- Somewhat against
- Strongly against

**End of Block**

**Start of Block: Improving understanding**

Und1.1 Please use the scale below to indicate how likely you think the following factors would help to improve public understanding of healthy nutrition.

|  | Very likely | Likely | Neither likely nor unlikely | Unlikely | Very unlikely |
| --- | --- | --- | --- | --- | --- |
| Improve the education that students receive |  |  |  |  |  |
| Improve scientific research |  |  |  |  |  |
| Improve media and news coverage |  |  |  |  |  |

Und2.1 Please use the scale below to indicate how likely you think the following factors would help improve public understanding of poverty.

|  | Very likely | Likely | Neither likely nor unlikely | Unlikely | Very unlikely |
| --- | --- | --- | --- | --- | --- |
| Improve the education that students receive |  |  |  |  |  |
| Improve scientific research |  |  |  |  |  |
| Improve media and news coverage |  |  |  |  |  |

Und3.1 Please use the scale below to indicate how likely you think the following factors would help improve public understanding of climate change.

|  | Very likely | Likely | Neither likely nor unlikely | Unlikely | Very unlikely |
| --- | --- | --- | --- | --- | --- |
| Improve the education that students receive |  |  |  |  |  |
| Improve scientific research |  |  |  |  |  |
| Improve media and news coverage |  |  |  |  |  |

Q71 Please use the scale below to indicate how likely you think the following factors would help improve public understanding of the effectiveness of medical drugs.

|  | Very likely | Likely | Neither likely nor unlikely | Unlikely | Very unlikely |
| --- | --- | --- | --- | --- | --- |
| Improve the education that students receive |  |  |  |  |  |
| Improve scientific research |  |  |  |  |  |
| Improve media and news coverage |  |  |  |  |  |

**End of Block: Improving understanding**

**Start of Block: Demographics**

age What is your age?

|  | 0 | 10 | 20 | 30 | 40 | 50 | 60 | 70 | 80 | 90 | 100 |
| --- | --- | --- | --- | --- | --- | --- | --- | --- | --- | --- | --- |

| Years old | 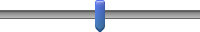 |
| --- | --- |

sex What is your sex?

- Female
- Male
- Other

race With what ethnic group do you associate best?

- Native American
- Asian
- Black, African American or African European
- Hispanic, Latino, or South American
- Arab
- White
- Other

nation What is your nationality?

▼ Afghanistan ... Zimbabwe

education What is the highest level of formal education you attained?

- No education
- Primary/elementary school
- Secondary/middle school
- University/College undergraduate degree
- University/College postgraduate degree
- PhD or MD

*urban rural To the best of your knowledge, do you live in a more urban or rural area?*

- More urban area - with more than 100,000 inhabitants
- More rural area - with less than 100,000 inhabitants

academic background If you are presently a student or academic member at a higher education institution and/or have already completed a degree at a higher education institution, what subject area best applies to what you study, work in or completed your degree in

- Natural science
- Social science
- Arts and humanities
- Not applicable

job Which of the following job titles best applies to you?

- Primary sector - including agriculture, mining and other natural resource industries
- Secondary sector - including manufacturing, engineering and construction
- Tertiary sector - service industry
- Other

vote Did you vote in the last national elections in your country?

- Yes
- No

politics What is your political orientation?

- Liberal
- Conservative
- None
- Other

news Which source do you use most for receiving news?

- Television
- Radio
- Newspaper
- Social media
- Other

religion What is your religious affiliation?

- Muslim
- Catholic
- Christian, non-Catholic
- Jewish
- Hindu
- Buddhist
- Other
- None

income What is your current level of annual income?

- Up to 10,000 US Dollars
- 10,000 – 20,000 US Dollars
- 20,000 – 40,000 US Dollars
- 40,000 – 80,000 US Dollars
- More than 80,000 US Dollars

End of survey.

Comment. If you may have a comment on a question you answered in this study you are welcome to share it here:

________________________________________________________________

Thanks for your time and participation!

**End of Block: Demographics**
