# Supplementary material for: Correction: Explaining public understanding of the concepts of climate change, nutrition, poverty and effective medical drugs: An international experimental survey
Source: PLoS One. 2020 Aug 5;15(8):e0237445. doi: 10.1371/journal.pone.0237445 (PMC7406070; doi:10.1371/journal.pone.0237445)
Supplement: S1 File — (PDF) [file pone.0237445.s001.pdf]

PLOS ONE

RESEARCH ARTICLE

Explaining public understanding of the concepts of climate change, nutrition, poverty and effective medical drugs: An international experimental survey

Alexander Krauss[1,2]*, Matteo Colombo[3]

1 London School of Economics, London, United Kingdom, 2 University of Barcelona, Barcelona, Spain, 3 Tilburg University, Tilburg, the Netherlands

* a.krauss@lse.ac.uk

Check for updates

OPEN ACCESS

Citation: Krauss A, Colombo M (2020) Explaining public understanding of the concepts of climate change, nutrition, poverty and effective medical drugs: An international experimental survey. PLoS ONE 15(6): e0234036. https://doi.org/10.1371/journal.pone.0234036

Editor: Isabel Novo-Cortí, Universidade da Coruna, SPAIN

Received: February 9, 2020

Accepted: May 15, 2020

Published: June 10, 2020

Copyright: © 2020 Krauss, Colombo. This is an open access article distributed under the terms of the Creative Commons Attribution License, which permits unrestricted use, distribution, and reproduction in any medium, provided the original author and source are credited.

Data Availability Statement: Data are available as Supporting Information.

Funding: This research was funded through the Marie Curie programme of the European Commission and the Beatriu de Pinós programme of the Government of Catalonia (Alexander Krauss) and through an Alexander von Humboldt research fellowship (Matteo Colombo). The funders had no role in study design, data collection and analysis, decision to publish, or preparation of the manuscript.

Abstract

Climate change, nutrition, poverty and medical drugs are widely discussed and pressing issues in science, policy and society. Despite these issues being of great importance for the quality of our lives it remains unclear how well people understand them. Specifically, do particular demographic and socioeconomic factors explain variation in public understanding of these four concepts? To what extent are people's changes in understanding associated with changes in their behaviour? Do people judge scientific practices relying on the more descriptive concepts of *climate change* and *effective medical drugs* to be more objective (less controversial) than practices relying on the more value-laden concepts of *poverty* and *healthy nutrition*? To address these questions, an experimental survey and regression analyses are conducted using data collected from about one thousand participants across different continents. The study finds that public understanding of science is generally low. A smaller proportion of people were able to correctly identify the common explanation accepted internationally among the scientific community for climate change and effectiveness of medical drugs (42% and 43% of participants in the study, respectively) than for poverty and healthy nutrition (61% and 65% of participants, respectively). Older age and political non-conservativeness were the strongest predictors of correctly understanding these four concepts. Greater levels of education and political non-conservativeness were in turn the strongest predictors of people's reported changes in their behaviour based on their improved understanding of these concepts. Because climate change is among the least understood scientific concepts but is arguably the greatest challenge of our time, better efforts are needed to improve how media, awareness campaigns and education systems mediate information on the topic in order to tackle the large knowledge deficits that constrain behavioural change.

Competing interests: The authors have declared that no competing interests exist.

1. Introduction

Existing research in psychology, philosophy, social sciences and communication sciences has examined how people understand scientific concepts and the role and significance of values in scientific practice, as well as their consequences for the objectivity of science and for public trust in science [1,2,3,4,5,6]. Specifically, research on public understanding of science has focused on individual scientific domains in isolation–such as climate science or nutrition–examining how scientific understanding, political convictions and personal values can influence public perception of science, or predict people's behaviour [7,8,9,10,11,12,13,14,15,16].

Most people understand what *climate change* is on the basis of subjective experiences such as of increasingly irregular meteorological patterns, knowledge of facts like global warming, and individual trust in different sources of information and social actors [17]. In particular, the term 'global warming' has been found to evoke more concern than 'climate change' [18]. While levels of education may not always predict levels of scepticism and concern about climate change [19,20], public engagement on climate change is reliably predicted by individuals' liberal values [10,12,7].

In terms of *healthy nutrition*, reliable predictors of people following a healthy diet include their knowledge of nutrition, level of education, local food culture and higher socio-economic status [21,22,23,24,25]. People in Europe and North America for example have been found to understand this concept in terms of specific foods, such as fruits and vegetables [26,27], their origin such as organic versus conventional foods [28], and features of a healthy diet, like balance, variety and moderation [29,30].

Across different countries and demographic backgrounds, significant variation has also been observed in public understanding of what *poverty* means, of the causes and consequences of living in poverty, and of the circumstances under which the poor deserve public assistance [31,32,33]. According to the EU Poverty and Social Exclusion Survey [34], about 25% of European citizens believe that to be poor is to lack material resources necessary for full participation in society, while 21% believe that to be poor is to be dependent on public subsidies or charity.

Existing research on the concept of *effective medical drugs* focuses in turn on public views about the efficacy of alternative treatments like homeopathy or of "generic drugs" in comparison to standard medical drugs [35,36,37]. This research highlights that the vast majority of the public has little or no knowledge of actual medical and pharmaceutical research [38]. Although the most important factors predicting patients' adherence to treatment are personal beliefs about efficacy and safety [39,40], it remains unclear how patients define and explain the efficacy of a drug (cf. [41]).

This experimental study contributes to this body of research by identifying the demographic, ideological (political and religious) and epistemic factors that can explain differences in public understanding of four of the most commonly discussed scientific concepts, *climate change*, *healthy nutrition*, *poverty* and *effective medical drugs*. The study was designed to address three main questions. First, what are the demographic, ideological and epistemic factors that most reliably explain variation in people's understanding of *climate change*, *healthy nutrition*, *poverty* and *effective medical drugs*? Second, are people's changes in their understanding of these four concepts associated with changes in their behaviour? Third, do people judge scientific practices employing the more descriptive concepts of *climate change* or *effective drugs* to be more objective (less controversial) than practices employing the concepts of *poverty* or *nutrition*, which are both descriptive and evaluative [42] at the same time?

Corresponding to these three questions, and building on the results of the extant research outlined above, we formulated three main hypotheses prior to conducting the study. First, across the different study samples and conditions, survey participants' correct understanding

of all four concepts, *climate change*, *healthy nutrition*, *poverty* and *effective medical drugs*, is most reliably predicted by their higher socio-economic status, liberal values, and existing knowledge of the issue. Second, across the different study samples and conditions, survey participants' changes in their knowledge of an issue are associated with changes in their behaviour. Third, across the different study samples and conditions, survey participants judge the concepts of *climate change* and *effective drugs* to be more objective compared to the concepts of *poverty* and *nutrition*.

In testing these hypotheses, this experimental study advances existing literature in the intersection of psychology, philosophy and the social sciences in five novel ways. It assesses public understanding and behaviour related to four widely discussed value-laden scientific concepts comparatively, within the same study. It employs a unique set of controls that includes three experimental groups to better assess variation in results. It takes into account a broader range of predictor variables than similar studies to assess their relative importance and better assess the role of values. It evaluates how people across different cultures understand and behave differently on these issues (with three study samples from India, the US, and the rest of the world). And it probes the extent to which people's changes in behaviour are associated with changes in their understanding of these concepts.

Overall, the study indicates that knowledge deficits and ideological differences are the most powerful explanatory factors driving differences across cultural contexts in the motivation to change one's behaviour on the basis of their understanding of science. The findings are important both scientifically, for clarifying patterns of variation in public understanding of science, and practically, for addressing these issues, building effective cooperation between science and society, and better aligning scientific practice and its outcomes with the expectations, understanding and values of the public.

2. Methods and study design

The study collected data from 918 participants distributed across three study populations from India (29.4%), the US (41.6%) and the rest of the world (29%)–which includes participants from 52 countries, with the largest shares from Brazil, Italy, Germany, the UK and Canada. Participants were recruited in October 2019 using Amazon Mechanical Turk (MTurk). For the study population, participants were randomly assigned to one of three experimental groups. In experimental group 1, participants answered a set of questions about what they viewed as the scientific community's views on climate change, healthy nutrition, poverty and effective medical drugs. In experimental group 2, participants answered the identical questions, but focused on their own views about the four issues. (For questions where it is not feasible or directly relevant to ask about the scientific community's views or participants' views, then the general public's or the government's views on these issues were studied.) In experimental group 3, participants answered both sets of questions. The study thus adopted a mixed experimental method that employed both a between-subjects design (experimental groups 1 and 2) and a within-subjects design (experimental group 3).

The survey questionnaire we developed included three sets of questions. The first set of questions aimed at eliciting participants' judgements about their understanding of climate change, healthy nutrition, poverty and effective medical drugs. The second set of questions aimed at eliciting participants' judgments about their behaviour and motivations in relation to these four issues. The third set of questions concerned participants' demographic and socio-economic characteristics, and cultural and political values.

To test the main hypotheses, we designed the survey questions and response items building on existing studies on the public's understanding of climate change, healthy nutrition, poverty

and effective medical drugs [20,7,39,19]. Understanding is viewed here as the ability to correctly define and explain a given concept. By correct definitions and explanations we mean those adopted by the leading international institutions in the relevant domain, namely: UNFCC for *climate change* [43,44], World Health Organisation for *nutrition* [45], World Bank for *poverty* [46,47] and Cochrane Collaboration for *medical drug effectiveness* [48,49]. For most questions in the survey, we apply a five-point Likert scale–the most commonly used scale in survey research. We also asked five experts, holding PhDs in psychology, philosophy and economics, to validate the survey questionnaire. After revision and validation we carried out the survey questionnaire, which is available in the supporting information (S1 File). We obtained approval on the survey from the Ethics, Data Management and Protection board of the School of Humanities and Digital Sciences at Tilburg University (The Netherlands).

In terms of the representativeness of MTurk participants, results from surveys indicate that they 'generally provide high-quality data and are reasonably representative of the general population across most psychological dimensions assessed' [50] cf. [51]. For the study's overall sample, background traits of participants are well represented–though females can be underrepresented in Mturk samples [52,51]–with 32% of all participants in the survey female and 68% male. The average age of participants was 34 years (see last column of Table 3 for full demographics). Participants received a payment of $1.50 for completing the survey, which is comparable with payments for completing academic surveys of similar length via Mturk [52,51].

Regression analysis was conducted using three dependent variables, namely: participants' ability to *define* and *explain* (and thus understand) the four target concepts, and participants' motivation to change their *behaviour* on the basis of their understanding. These three variables were studied, while controlling for participants' demographics, socioeconomic status, education, and political and religious values (i.e. the independent variables).

In terms of measuring people's political/ideological values, different scales exist that each has methodological strengths and weaknesses [53,54]. This survey allowed participants to self-identity in terms of liberal or conservative political orientation. We apply this distinction based on several considerations–though there can be ambiguity in using these terms in some languages. Our choice coheres with several researchers in social psychology relying on respondents' self-identification in terms of liberal and conservative [55]. There is, across countries, overlap between the meaning of left vs. right, and liberal vs. conservative, which can be useful for international comparisons; the distinctions are 'ubiquitous [in politics and] public opinion surveys all over the world, self-placement on a left–right scale stands out as something of a "super-issue," which "tends to assimilate all important issues" and consistently proves to be one of the best predictors of a person's political attitudes and behaviour' [56].

3. Results and discussion

The three main results of the study are the following. First, participants' understanding of *climate change*, *healthy nutrition*, *poverty*, and *effective medical drugs* is limited. In particular, older age and liberal political values were the strongest predictors of correctly understanding the four concepts. Second, greater levels of education and liberal political values were the strongest predictors of participants' reporting that they changed their behaviour based on their improved understanding. Third, respondents had an exaggerated belief that they could define and explain the four concepts better than other people, and they understood the concepts of *climate change* and *effective medical drugs* less accurately than *poverty* and *healthy nutrition*.

3.1 Differences in public understanding of value-laden science

Table 1 illustrates the descriptive results, which allow for comparing the relative differences in participants' ability to define and explain these four scientific concepts, and motivation to change behaviour based on their understanding.

To assess people's ability to *define* these concepts, respondents were asked the following question (for example, on nutrition): *Which one of these possible definitions of healthy nutrition do you believe is the most widely accepted within the scientific community*? *(i) Healthy nutrition is the result of eating any food in whatever amount that makes one feel good. (ii) Healthy nutrition is the result of eating a variety of foods, plenty of vegetables and fruit, moderate amounts of fats and oils and less salt and sugars. (iii) Healthy nutrition is the result of eating only vegetables, fruit, legumes, mushrooms and nuts, and avoiding all animal foods including milk and honey*. The second response is the correct definition as adopted by the World Health Organisation [45]. Questions and responses, which followed the same structure, were applied for all four issues (see S1 File survey questionnaire, for more details).

The share of participants who correctly identified which *definition*, among the three possible options, was the most widely accepted within the scientific community ranged from 72% for healthy nutrition, 66% for effective medical drugs, and 65% for climate change, to 53% for poverty (Table 1). Descriptive statistics illustrate that participants of older age, employed outside of agriculture, more politically liberal rather than conservative, wealthier and female, were more likely to identify the correct definition across all four topics. Participants from India were about half as likely to correctly define the four target concepts compared to individuals from the US or the rest of the world.

To assess people's ability to *explain* these concepts, respondents were asked the following question (for example, again, on nutrition): *Which one of these possible explanations of what makes nutrition healthy do you believe is the most widely accepted within the scientific community*? *(i) Healthy nutrition is how we defend ourselves against disease and aging. Poor nutrition can lead to lower bodily functions, and reduced levels of physical output and effectiveness. (ii) Healthy nutrition is how we acquire energy and the building blocks for our bodily system. Poor nutrition can lead to inadequate energy, and low mental functioning and efficiency. (iii) Healthy nutrition is how we attain good health through an adequate, well balanced diet. Poor nutrition can lead to reduced immunity, increased susceptibility to disease, impaired physical and mental development, and reduced productivity*. The third response is the correct explanation (World Health Organisation [45]; see S1 File survey questionnaire). The share of respondents who identified the correct *explanation* was lower for the less value-laden concepts of *climate change* and *effective medical drugs*, at 42% and 43% of participants, than for *healthy nutrition* and *poverty*, at 65% and 61% of participants. Descriptive statistics illustrate that older individuals and politically non-conservative individuals were more likely to identify the correct explanation on all four topics.

These results indicate that while scientific and political consensus exists on these four concepts–as reflected by leading international institutions (UNFCC [43,44], World Health Organisation [45], World Bank [46,47], Cochrane Collaboration [48,49]–public understanding lags behind, with less than half of participants able to explain what determines climate change and the effectiveness of a medical drug.

In terms of self-reported behavioural change, the vast majority of participants answered affirmatively to the question: *Has your understanding of the issue under consideration improved in the past 10 years*? Among this share of participants, over 70% reported that, across all four issues, *their behaviour changed in the past 10 years based on their new knowledge*. In particular, participants, who had greater levels of education and who were politically more liberal than

Table 1. Descriptive data: People's understanding and behaviour in relation to climate change, healthy nutrition, poverty and effective medical drugs–across their background traits and conditions.

	Share of participants correctly identifying which *definition* is the most widely accepted within the scientific community				Share of participants correctly identifying which *explanation* is the most widely accepted within the scientific community				Total # of participants	Share of participants who changed their *behaviour* due to improved knowledge on:				Total # of participants
	climate change	healthy nutrition	poverty	effective-ness of medical drugs	climate change	healthy nutrition	poverty	effective-ness of medical drugs		climate change	healthy nutrition	poverty	effective-ness of medical drugs	
Total	***65***	***72***	***53***	***66***	***42***	***65***	***61***	***43***	***612***	***78***	***86***	***74***	***75***	***851***
Female	67	78	57	74	41	68	60	44	196	80	91	71	75	262
Male	64	69	52	62	42	64	61	43	414	77	83	74	74	586
Age 18–29	56	61	50	56	37	57	56	37	241	82	85	73	76	348
Age 30–39	71	75	54	70	41	68	64	44	254	78	86	77	76	339
Age 40 >	71	88	61	77	52	75	65	53	117	71	88	68	68	164
Nationality														
US	74	82	60	75	41	71	65	48	258	76	85	64	70	332
India	40	43	33	42	35	45	48	34	173	83	88	83	83	267
Rest of World	76	84	64	75	49	76	68	46	181	76	84	73	70	252
Urban (more than 100,000 inhab.)	63	69	52	64	40	64	59	42	420	80	86	76	76	599
Rural	70	77	57	71	45	68	66	46	192	74	85	66	72	252
Highest level of education														
Secondary school	85	88	67	79	44	74	71	50	82	66	79	60	60	116
University undergraduate degree	62	69	53	62	40	64	62	44	378	79	86	74	75	529
University postgraduate degree or higher	61	71	47	69	43	63	53	38	150	81	89	78	80	200
Occupation (sector)														
Primary (e.g. agriculture)	48	52	29	48	39	46	56	46	95	81	89	78	76	149
Secondary (e.g. engineering)	58	61	44	62	36	60	56	43	206	80	85	77	76	284
Tertiary (e.g. service industry)	72	83	63	71	45	75	65	38	192	74	84	63	70	265
Other	80	88	74	79	46	73	67	50	119	80	87	80	78	153
Level of annual income (currently)														
Up to 10,000 US Dollars	62	73	59	63	41	67	57	41	104	81	84	81	79	158
10,000–20,000 US Dollars	55	64	43	59	43	58	62	46	158	80	87	81	79	200

(*Continued*)

Table 1. (Continued)

	Share of participants correctly identifying which *definition* is the most widely accepted within the scientific community				Share of participants correctly identifying which *explanation* is the most widely accepted within the scientific community				Total # of participants	Share of participants who changed their *behaviour* due to improved knowledge on:				Total # of participants
	climate change	healthy nutrition	poverty	effective-ness of medical drugs	climate change	healthy nutrition	poverty	effective-ness of medical drugs		climate change	healthy nutrition	poverty	effective-ness of medical drugs	
20,000–40,000 US Dollars	72	68	60	67	36	66	62	39	183	77	85	70	74	272
More than 40,000 US Dollars	69	83	53	72	47	70	61	47	167	76	87	64	68	221
Political orientation														
Liberal	74	78	59	71	42	71	63	44	263	82	90	74	79	371
Conservative	52	59	40	56	37	55	55	42	219	76	82	74	74	314
None	67	79	64	73	49	69	67	43	95	73	81	67	63	119
Other	71	83	69	71	43	77	66	46	35	76	91	79	73	47
Voted in last elections														
Yes	64	70	52	64	40	64	60	41	521	79	87	74	76	737
No	69	81	60	74	52	74	64	54	91	69	77	73	66	114
Religious affiliation														
Muslim	56	75	47	47	38	69	63	47	32	68	83	77	67	35
Catholic	70	79	46	77	39	66	64	36	122	87	84	76	82	172
Christian, non-Catholic	65	73	62	63	35	67	61	44	98	74	88	73	76	138
Hindu	39	40	34	44	36	46	46	38	149	81	87	81	79	213
Other	68	89	79	89	42	68	53	42	19	90	85	68	82	27
None	84	89	67	78	52	77	72	53	181	73	85	63	61	250
News source most used														
Television	61	65	50	58	41	64	53	42	142	79	91	74	81	201
Radio	62	81	62	73	38	62	81	50	26	81	85	69	59	33
Newspaper	61	55	48	61	46	54	59	35	82	75	87	72	77	116
Social media	63	73	54	65	37	64	61	42	291	80	84	74	74	402
Other	87	96	63	89	56	87	73	59	71	73	82	75	70	99

https://doi.org/10.1371/journal.pone.0234036.t001

Table 2. People's perception and actual ability to define and explain, and change their behaviour, related to climate change, healthy nutrition, poverty, and medical drug effectiveness.

		Ex-ante perception	Testing people's actual ability		Ex-post perception			Behavioural change	
		'I' / The 'general public' can explain what it is that makes/ causes climate change/ nutrition healthy/ poverty/a medical drug effective?	Which definition of x do you believe is the most widely accepted within the scientific community/ general public?	Which explanation of x do you believe is the most widely accepted within the scientific community/ general public?	Think again about your knowledge of x. Do you agree that you can (still) explain what x is?	Before-and-after difference between perception of being able to explain	*Total # of respondents*	Change in behaviour, among people who stated their understanding on 'x' improved in the past 10 years and were able to correctly define as well as explain 'x'	*Total # of respondents*
		%who agrees	% who identified correct definition	% who identified correct explanation	% who still believe they can explain				
		i			ii	ii–i =			
Climate change									
Experimental group 1	scientific community	. . .	64	43	. . .	. . .		. . .	. . .
	general public	50	. . .	. . .	. . .	. . .	*301*	. . .	. . .
	I				69	. . .		81	*83*
Experimental group 2	general public	. . .	65	42	. . .	. . .	*306*	. . .	. . .
	I	77	. . .	. . .	75	-2.3		81	*79*
Experimental group 3	scientific community	. . .	66	41	. . .	. . .	*311*	. . .	. . .
	general public	52	52	40	. . .	. . .		. . .	. . .
	I	71	. . .	. . .	70	-1.3		78	*77*
Healthy nutrition									
Experimental group 1	scientific community	. . .	73	68	. . .	. . .		. . .	. . .
	general public	59	. . .	. . .	. . .	. . .	*301*	. . .	. . .
	I				67	. . .		86	*155*
Experimental group 2	general public	. . .	74	60	. . .	. . .	*306*	. . .	
	I	79	. . .	. . .	82	3.0		81	*143*
Experimental group 3	scientific community	. . .	71	63	. . .	. . .	*311*	. . .	. . .
	general public	60	57	50	. . .	. . .		. . .	. . .
	I	75	. . .	. . .	77	2.6		93	*142*
Poverty									

(*Continued*)

Table 2. (Continued)

		Ex-ante perception	Testing people's actual ability		Ex-post perception			Behavioural change	
		'I' / The 'general public' can explain what it is that makes/ causes climate change/ nutrition healthy/ poverty/a medical drug effective?	Which definition of x do you believe is the most widely accepted within the scientific community/ general public?	Which explanation of x do you believe is the most widely accepted within the scientific community/ general public?	Think again about your knowledge of x. Do you agree that you can (still) explain what x is?	Before-and-after difference between perception of being able to explain	*Total # of respondents*	Change in behaviour, among people who stated their understanding on 'x' improved in the past 10 years and were able to correctly define as well as explain 'x'	*Total # of respondents*
		%who agrees	% who identified correct definition	% who identified correct explanation	% who still believe they can explain				
Experimental group 1	scientific community	. . .	60	62	. . .	. . .		. . .	. . .
	general public	54	. . .	. . .	. . .	. . .	*301*	. . .	. . .
	I				79	. . .		70	*84*
Experimental group 2	general public	. . .	53	49	. . .	. . .	*306*	. . .	. . .
	I	74	. . .	. . .	82	8.2		68	*68*
Experimental group 3	scientific community	. . .	47	60	. . .	. . .	*311*	. . .	. . .
	general public	53	42	42	. . .	. . .		. . .	. . .
	I	66	. . .	. . .	76	10.0		73	*41*
Effective medical drugs									
Experimental group 1	scientific community	. . .	66	55	. . .	. . .		. . .	. . .
	general public	29	. . .	. . .	. . .	. . .	*301*	. . .	. . .
	I				63	. . .		69	*68*
Experimental group 2	general public	. . .	64	43	. . .	. . .	*306*	. . .	. . .
	I	44	. . .	. . .	62	18.0		68	*59*
Experimental group 3	scientific community	. . .	66	32	. . .	. . .	*311*	. . .	. . .
	general public	35	47	55	. . .	. . .		. . .	. . .
	I	44	. . .	. . .	63	19.0		71	*42*

https://doi.org/10.1371/journal.pone.0234036.t002

conservative, were the most likely to report they changed their behaviour based on their improved understanding. Participants with a university postgraduate degree or higher were about 20% more likely to have changed their behaviour related to climate change, poverty and medical drugs compared to those with only secondary school education. Age was also associated with participants' reporting that they changed their behaviour on the basis of their improved understanding, with younger individuals more likely to change their behaviour after learning about the particular issue. Finally, while participants with higher levels of income

Table 3. Estimated marginal effects of the traits and conditions of people that predict their ability to correctly *define* climate change, healthy nutrition, poverty and effective medical drugs.

	Model 1								Model 2								*Descriptive data avg. (% of group out of 100)*
**Dependent variable:*(1 if defined correctly, 0 if not)***	**Climate change**		**Healthy nutrition**		**Poverty**		**Effectiveness of medical drugs**		**Climate change**		**Healthy nutrition**		**Poverty**		**Effectiveness of medical drugs**		
Independent variables (all variables 1 or 0)	Coef.	T-stat	Coef.	T-stat	Coef.	T-stat	Coef.	T-stat	Coef.	T-stat	Coef.	T-stat	Coef.	T-stat	Coef.	T-stat	
Female (reference group, male)	-0.00414	-0.0965	0.0645*	1.705	0.0301	0.677	0.0960**	2.354	-0.0495	-1.017	0.0230	0.564	-0.0366	-0.719	0.0930**	2.114	*0.32*
Age 30–39 (ref. group, 18–29)	0.131***	3.134	0.0999***	2.727	0.0312	0.677	0.109***	2.646	0.123***	2.770	0.0769**	2.000	-0.00566	-0.110	0.0826*	1.858	*0.42*
Age 40 >	0.102**	2.033	0.201***	5.453	0.0880	1.521	0.152***	3.219	0.0444	0.755	0.159***	4.108	0.00178	0.0266	0.106**	1.969	*0.19*
Urban (ref. group, rural)	-0.0165	-0.372	-0.0398	-1.000	0.00308	0.0663	-0.0451	-1.046	-0.0244	-0.509	-0.0540	-1.340	0.00164	0.0317	-0.0507	-1.106	*0.69*
Education: Completed secondary (ref. group, university postgraduate+)	0.239***	4.919	0.148***	2.963	0.197***	3.085	0.0844	1.276	0.163***	2.656	0.0151	0.208	0.0986	1.256	-0.0276	-0.337	*0.13*
Completed university undergraduate degree	0.0342	0.733	-0.0121	-0.287	0.0803	1.613	-0.0664	-1.447	-0.00426	-0.0867	-0.0530	-1.288	0.0643	1.160	-0.110**	-2.332	*0.62*
Annual income: Up to 10,000 USD (ref. group, > 40,000 USD)	-0.0724	-1.127	-0.0868	-1.294	0.0607	0.961	-0.0649	-1.001	-0.000272	-0.00390	-0.0129	-0.193	0.0786	1.077	0.00252	0.0364	*0.17*
10,000–20,000 USD	-0.130**	-2.263	-0.169***	-2.829	-0.0936	-1.631	-0.0887	-1.549	-0.0216	-0.349	-0.0309	-0.522	-0.0368	-0.555	0.00740	0.121	*0.26*
20,000–40,000 USD	0.0534	1.023	-0.144**	-2.557	0.0780	1.444	-0.0281	-0.520	0.115**	2.210	-0.0776	-1.403	0.119**	2.048	0.0202	0.368	*0.30*
Nationality: US (reference group, Rest of the world)									-0.0623	-1.095	-0.0232	-0.452	-0.0142	-0.244	0.00123	0.0225	*0.42*
Indian									-0.150	-1.490	-0.0882	-1.017	-0.243**	-2.409	-0.226**	-2.229	*0.28*
Occupational sector: Secondary—e.g. engineering (ref. group, primary—e.g. agriculture)									0.103*	1.846	0.0676	1.483	0.171**	2.568	0.139**	2.571	*0.33*
Tertiary—e.g. service industry									0.110*	1.876	0.153***	3.463	0.251***	3.904	0.113**	1.969	*0.31*
Other occupation									0.150**	2.400	0.132***	2.672	0.317***	5.002	0.114*	1.751	*0.19*

(*Continued*)

Table 3. (Continued)

	Model 1								Model 2								
**Dependent variable:*(1 if defined correctly, 0 if not)***	**Climate change**		**Healthy nutrition**		**Poverty**		**Effectiveness of medical drugs**		**Climate change**		**Healthy nutrition**		**Poverty**		**Effectiveness of medical drugs**		***Descriptive data avg. (% of group out of 100)***
Political orientation: Conservative (ref. group, liberal)									-0.164***	-3.257	-0.107**	-2.363	-0.145***	-2.781	-0.108**	-2.194	*0.36*
None									-0.165**	-2.202	-0.0268	-0.419	0.0372	0.530	-0.0220	-0.319	*0.16*
Other									-0.120	-1.067	0.0200	0.220	0.0510	0.486	-0.0131	-0.131	*0.06*
Voted in last elections, yes (ref. group, no)									0.0213	0.316	0.0137	0.214	0.0400	0.563	-0.00513	-0.0778	*0.85*
Religious affiliation: Christian, Catholic (ref. group, Muslim)									0.0445	0.541	0.00281	0.0353	-0.161*	-1.692	0.165**	2.440	*0.20*
Christian, non-Catholic									0.00325	0.0363	-0.0742	-0.788	0.0270	0.268	0.00507	0.0575	*0.16*
Hindu									-0.0941	-0.872	-0.185*	-1.721	-0.0111	-0.0943	0.102	1.131	*0.24*
Other									-0.0173	-0.122	0.0308	0.226	0.173	1.218	0.213**	2.525	*0.03*
None									0.146*	1.852	0.0111	0.134	-0.0226	-0.230	0.0969	1.216	*0.30*
News source most used: Television (ref. group, newspaper)									-0.0235	-0.324	0.0438	0.812	0.0249	0.319	-0.0502	-0.698	*0.23*
Radio									-0.0380	-0.308	0.134**	2.372	0.112	0.925	0.0824	0.826	*0.04*
Social media									-0.0432	-0.667	0.101*	1.931	0.0174	0.245	-0.000633	-0.0101	*0.48*
Other									0.100	1.163	0.211***	5.329	-0.0564	-0.586	0.192***	2.946	*0.12*
Number of observations	612		612		612		612		612		612		612		612		
R-squared	0.0552		0.0795		0.0277		0.0462		0.159		0.230		0.126		0.140		

*** $p<0.01$

** $p<0.05$

* $p<0.1$. T-stat indicates if a variable's significance level is over 95% if > 1.96 or < 1.96. Urban refers to geographic areas with more than 100,000 inhabitants.

https://doi.org/10.1371/journal.pone.0234036.t003

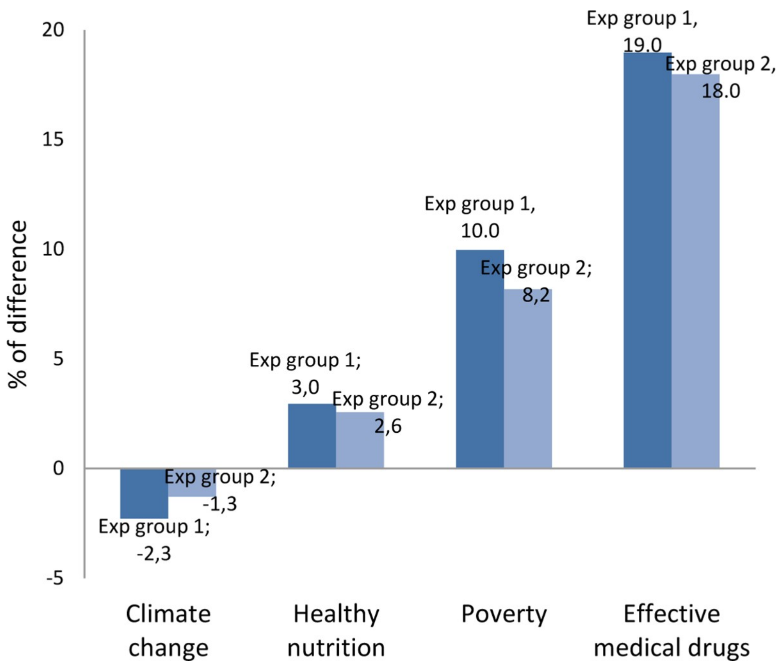

Fig 1. Share of difference in people's perception–before and after actually testing them–for being able to explain climate change, healthy nutrition, poverty, and medical drug effectiveness.

https://doi.org/10.1371/journal.pone.0234036.g001

were better able to define and explain all four concepts, they were less willing, compared to the rest of participants, to change their behaviour on the basis of their improved understanding of the issues of climate change, poverty, and medical drugs.

3.2 An "illusion of understanding" and a "better-than-average" effect in public understanding of value-laden science

Prior to conducting the study we expected that asking respondents whether they could identify the correct explanation for each of the four concepts would elicit an "illusion of understanding," whereby most participants would realize that they understand the world with far less detail, coherence and depth than they previously assumed [13,57,16].

To elicit this effect, participants were first asked to what extent they think they could explain a given scientific concept–climate change, for example. Afterwards, two further questions were asked: *Which one of three possible definitions of climate change do you believe is the most widely accepted within the scientific community*? and *Which one of three possible explanations of climate change do you believe is the most widely accepted within the scientific community*? After participants answered these questions, they were asked again to what extent they think they could (still) explain the target concept.

The share of participants, who initially stated that they could *explain* climate change or healthy nutrition was over 70%, for poverty over 60%, and for medical drugs' effectiveness over 40%. When testing people's ability to identify the correct definition and explanation, at least 63% of participants were able to do so for healthy nutrition, and 47% for poverty, and reduced to 41% for climate change and 32% for medical drugs' effectiveness. Unlike what we expected, the share of participants who then reported that they could still *explain* the target scientific concepts increased for healthy nutrition by 3% and 2.6% (for experimental groups 2 and 3, respectively), for poverty by 8.2% and 10%, and for effective medical drugs by 18% and

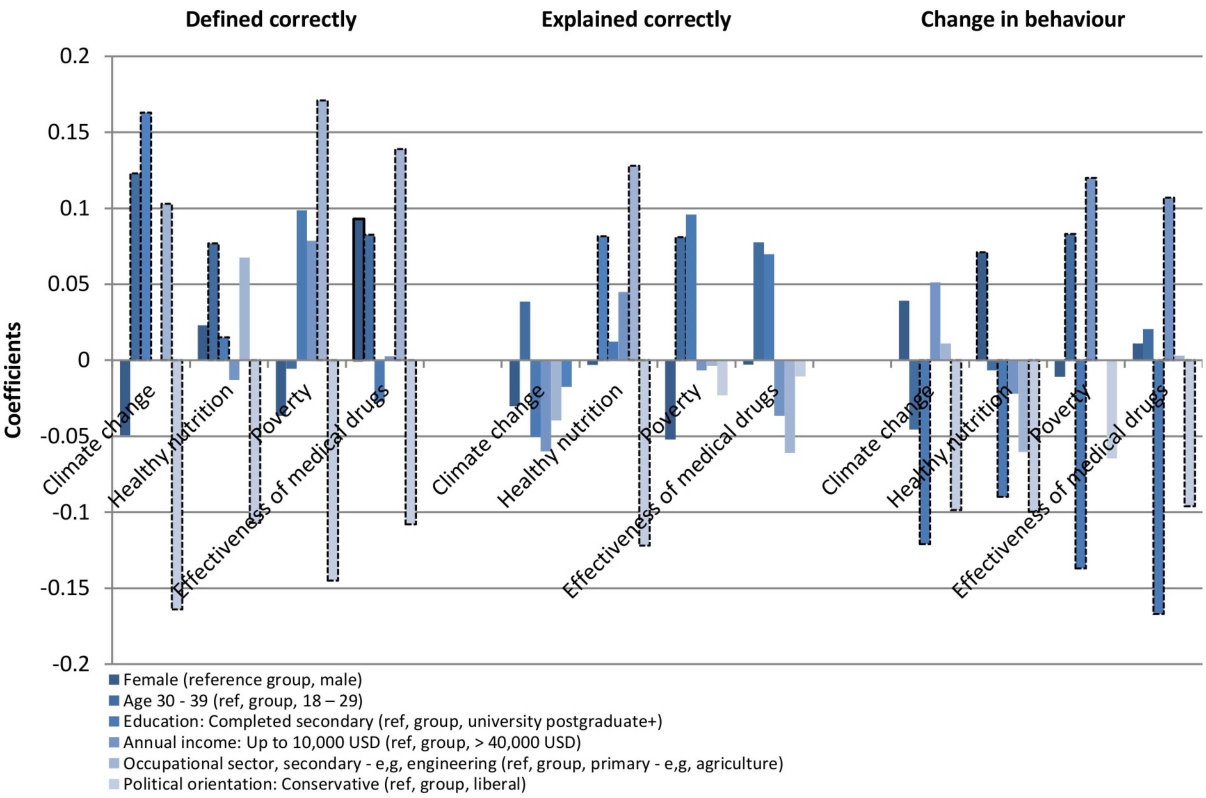

Fig 2. Estimated marginal effects of the traits and conditions of people that predict their ability to correctly define and explain, and change their behaviour, related to climate change, healthy nutrition, poverty and effective medical drugs. Statistically significant coefficients at p<0.1 or higher are reflected with a black dashed border.

https://doi.org/10.1371/journal.pone.0234036.g002

19%. Only for climate change was an effect found that is compatible with an illusion of understanding, since participants' confidence that they could explain this concept decreased by 2.3% and 1.3% after receiving the questions (for experimental groups 2 and 3, respectively).

Prior to conducting the study we also expected that participants would demonstrate a "better-than-average" effect, whereby they would rate their understanding of the target concepts as better than average [58]. The results supported this expectation, as participants both across and within the same experimental groups tended to report that they could explain all four concepts better than the general public. Among those unable to identify the correct definition or explanation associated with any of the four concepts, the share of participants still reporting that they could provide correct explanations was high, at 79% for poverty, 76% for climate change, 69% for healthy nutrition and 68% for medical drugs' effectiveness.

Among participants who stated that their knowledge on one of the four target concepts improved in the past 10 years, and who correctly understood that concept, about two thirds changed their behaviour after gaining new knowledge: at least 81% regarding nutrition, 78% regarding climate change and 68% regarding poverty and medical drug use (Table 2). Behavioural change is thus highly correlated with improved understanding.

Fig 1 visually represents the before-and-after differences in people's perception of whether they can explain these scientific concepts after having been tested on whether they actually could do so, with people, after being tested (and thus reading possible options), becoming more confident that they are able to–except in the case of climate change.

3.3 Predicting people's understanding and behaviour in the face of value-laden science

To identify predictors of people's ability to define and explain value-laden scientific concepts, as well as their willingness to change their behaviour (i.e. the dependent variables), two logistic regression models were employed. Model 1 controls for a common set of background factors that includes gender, age, geographic location (urban or rural), level of education, and level of income (i.e. the independent variables) (see also [20,7,39]). Whitmarsh [20] employs a similar set of variables and tests also for people's political orientation to measure their level of uncertainty about climate change. Model 2 extends these variables controlling for political orientation (similarly to Whitmarsh [20] and Hornsey *et al.* [7]) as well as nationality to assess cross-country variation, religious affiliation to test whether certain cultural values and ideology are associated with being more informed (similarly to Hornsey *et al.* [7]), occupation to test how people's type of job correlates with being more informed, political participation to test whether those who participate politically are more informed, and source of news to test whether the means through which people receive information influences how well they are informed (similarly to Brulle, Carmichael and Jenkins [19]). These controls have thereby been applied in different experimental designs to evaluate people's perceptions and understanding [7,20,19].

The estimated marginal effects of the logistic regression for Model 2 illustrates that older age, political non-conservativeness and non-agricultural occupations were the strongest predictors of participants' ability to correctly *define* the four scientific concepts under consideration (see Table 3). Nationality also predicted participants' ability to define these concepts, with participants in India less likely to be able to than participants in the US or those from the rest of the world.

Using the identical sets of controls and indicators (as in Table 3), regression analyses were then run to identify the most reliable predictors of participants' ability to *explain* these four scientific concepts, and of participants' willingness to *change their behaviour* based on new knowledge of these concepts. Older age was the strongest predictor of correct explanations; and political non-conservativeness and greater levels of education were consistently the strongest predictors of behavioural change.

To compare the results for the three dependent variables, Fig 2 visually illustrates the estimated marginal effects and the relative importance of six key background influencers on people's ability to correctly define and explain, and change their behaviour, related to these four concepts.

Fig 3 illustrates the disaggregated data by US and Indian populations. For participants from the US, liberal political orientation was the strongest and most consistent predictor of people's ability to identify correct *definitions*, while the effects of political orientation were much smaller and generally not statistically significant for participants from India and the rest of world. Older age predicted people's ability to define these concepts for the population from the US and the rest of world, but was not statistically significant for the Indian population.

While for the full sample (including all nationalities) the strongest and most consistent predictors of *behavioural change* were political non-conservativeness and greater levels of education, the disaggregated results illustrate that liberal political affiliation strongly predicts behavioural change among US participants and to a lesser extent among Indians. Completing more than secondary school education was a strong predictor for Indians' changing their behaviour. Females from the US were also more likely to change their behaviour than males, while in India the opposite was the case.

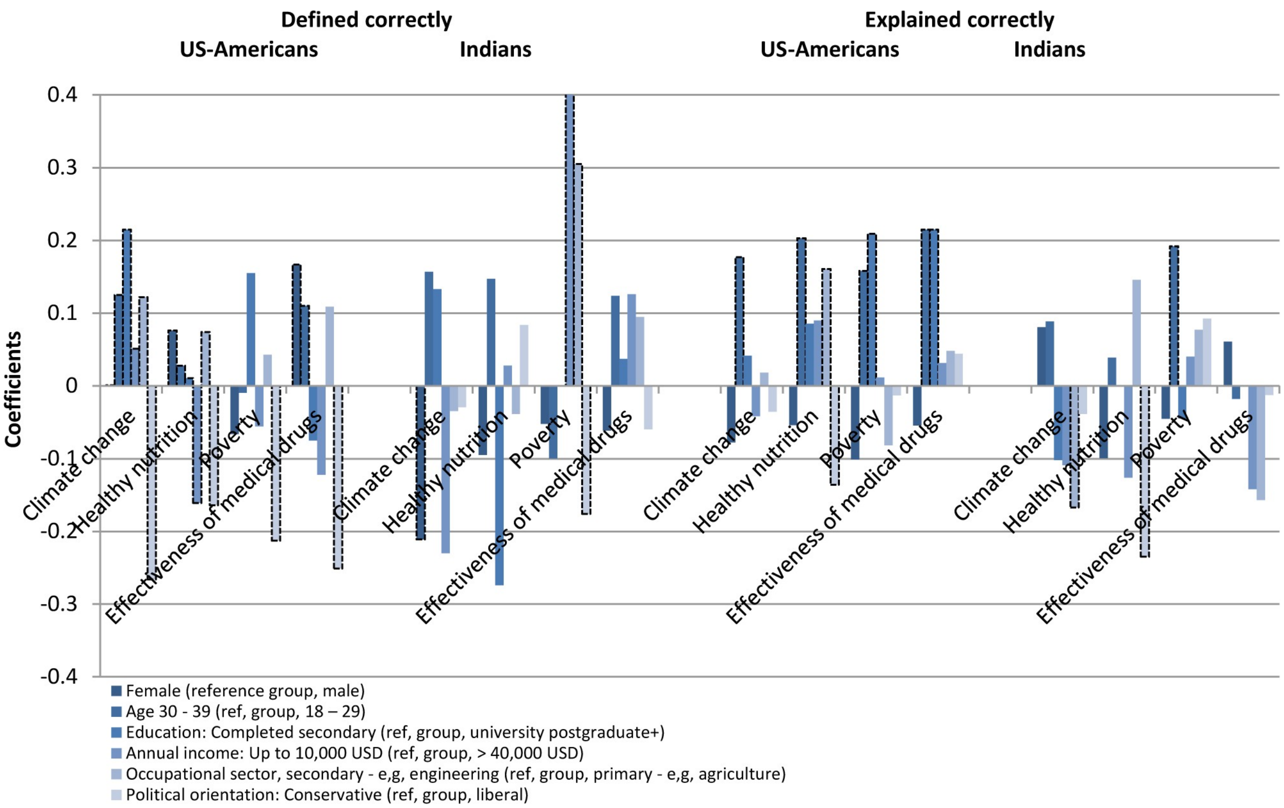

Fig 3. Estimated marginal effects of the traits and conditions of US-Americans and Indians that predict their ability to correctly define and explain climate change, healthy nutrition, poverty and effective medical drugs. Statistically significant coefficients at p<0.1 or higher are reflected with a black dashed border.

https://doi.org/10.1371/journal.pone.0234036.g003

3.4 Value-judgements, disagreement and objectivity in science

Table 4 illustrates that the vast majority of participants believed that both the scientific community and the government in their country can provide a definition of *climate change*, *healthy nutrition*, *poverty* and *effective medical drugs* without making value-judgements (that is, without appealing to somebody's opinions about what types of things are good or bad for someone). The same applies for providing an objective (that is, accurate) definition on these issues. These findings were consistent across the three experimental groups and four concepts. More evaluative scientific concepts like poverty or nutrition were thus not perceived as less accurate or reliable than more descriptive scientific concepts like climate change that figure in politically divisive public debates. Yet the evaluative dimension of poverty is essentially contestable, but it is not contestable that the climate is changing [59].

For those participants who believed there is scientific disagreement about how a given concept should be defined, they were asked about the reasons for the lack of agreement. Participants viewed *complexity of the topic* and *contradicting scientific studies on the topic* as the two strongest factors contributing to scientific disagreement.

3.5 Importance and responsibilities in improving public understanding of science

Participants were more inclined to believe that it is their own responsibility instead of the government's responsibility to ensure that the general public has an adequate understanding of

Table 4. Share of people who agrees that the scientific community and government can provide a value-free or objective definition of climate change, healthy nutrition, poverty and effective medical drugs.

		% who agrees the scientific community/government can provide a definition of 'x' without making value-judgements	% who agrees the scientific community/government can provide an objective (accurate) definition of 'x'	Total # of respondents *(918 in total)*
Climate change				
Experimental group 1	scientific community	70	79	301
Experimental group 2	government	59	64	306
Experimental group 3	scientific community	71	76	311
	government	61	61	
Healthy nutrition				
Experimental group 1	scientific community	72	80	301
Experimental group 2	government	67	72	306
Experimental group 3	scientific community	71	81	311
	government	67	71	
Poverty				
Experimental group 1	scientific community	65	71	301
Experimental group 2	government	71	74	306
Experimental group 3	scientific community	69	71	311
	government	63	68	
Effective medical drugs				
Experimental group 1	scientific community	70	73	301
Experimental group 2	government	69	71	306
Experimental group 3	scientific community	76	77	311
	government	68	67	

https://doi.org/10.1371/journal.pone.0234036.t004

science. Participants reported that the best way to improve such an understanding is through improving the education that students receive (with over 75% reporting this would lead to improvement), followed by improving media and news coverage (over 65%), and improving scientific research (over 60%).

Participants were also asked whether they were willing to pay 10% more in taxes, or on the price of relevant products, to implement policies to improve these issues. Participants were most willing to pay higher taxes for CO2 reducing measures (57% of all respondents), followed by reducing poverty (53%) and promoting healthy nutrition (47%), and finally promoting awareness on medical drugs (34%). About one in five participants stated that they were *strongly* in favour of paying more to improve these issues. Participants in group 1 (who were asked if they would be in favour of paying 10% more) compared to group 2 (who were asked if

they would be in favour of their government levying a 10% tax increase) consistently reported being more willing to pay more to improve all four issues. As the monetary effects on individuals would be identical in both experimental groups, this illustrates a framing effect and the importance of the way in which policymakers can formulate policies to improve these four issues.

4. Conclusion

This study examined public understanding and behaviour related to four scientific concepts simultaneously, comparatively and cross-culturally. It found that general understanding of these important scientific, environmental and social issues is overall low: with people able to correctly explain the concepts of *climate change* and *effective medical drugs* at 42% and 43%, and the concepts of *healthy nutrition* and *poverty* at 65% and 61%.

In relation to the main research questions about the demographic, ideological and epistemic factors that most reliably predict people's understanding of these four concepts, and about how changes in understanding are associated with changes in their behaviour, the study found that older age and non-conservative political values strongly predicted peoples' understanding on these four issues; it also found that greater levels of education and non-conservative political values strongly predicted changes in people's behaviour.

These results are consistent with our hypotheses about the predictive power of liberal values on increased public understanding of science, and about the motivational power of increased scientific understanding on changes in behaviour. The fact that no salient differences were found between the way survey participants judged the value-ladenness of, or degree of agreement about, the concepts of *climate change* and *effective drugs* on one hand, and *poverty* and *nutrition* on the other, indicates that the evaluative dimension of certain scientific concepts such as *poverty* need not be essentially contestable. The political and ideological context in which a 'non-evaluative' scientific concept like *climate change* is discussed can suffice to render it contestable.

The present study faces several limitations. One limitation is precisely this gap between one of our hypotheses–namely, that people may judge the more descriptive concepts of *climate change* and *effective drugs* to be more objective than the concepts of *poverty* and *nutrition*–and the study's evidence illustrating similar perceived levels of objectivity of these four concepts (Table 4). Though, most would agree that the concept of poverty is associated with greater value-ladenness in a way that the concept of climate change is not. The only explanation we can provide is that climate change is the most politicised among the four issues, with how people perceive climate change shaped by the political environment that may reduce its perceived objectivity. Another limitation is the focus on *self-reported* changes in people's behaviour (rather than using an *independent* measure). Some participant responses may be inaccurate due to limitations in recalling past changes in their behaviour. An extension of this study that would also include data about participants' behaviour, such as changes in their household energy consumption to reduce their carbon footprint, would help enrich our understanding of behavioural change by providing an additional measure and additional evidence. Another limitation is that results could only be compared between sample populations from the US, India and the rest of the world, while further country comparisons would provide a richer understanding of variations across cultures. Though this may require a more elaborate description in the survey of what is meant by liberal vs. conservative, and left vs. right, political orientation, so that data can be compared across countries.

Despite such limitations facing studies like ours, overall the study indicates that a deficit in knowledge and ideological differences are the most powerful factors explaining people's (lack

of) understanding of science and their unwillingness to change their behaviour on the basis of new knowledge. These results provide support to the "deficit model" of public understanding of science [60,61]. According to this model and in line with the present results, poor scientific literacy, together with non-liberal ideological orientation, can lead the public–for polarised scientific issues–to mistrust scientific institutions and evidence. Improving present low levels of public understanding, including on how political and ideological values can influence us [62], will be essential to foster informed behavioural change on these important value-laden scientific issues that affect our lives and (in the case of climate change) also our planet. Addressing these four issues and the associated knowledge deficits requires however greater efforts to improve how media, awareness campaigns and education systems mediate information on these pressing issues.

Supporting information

S1 File. Survey questionnaire.
(DOCX)

S2 File. Dataset, raw.
(DTA)

Acknowledgments

We are thankful to survey respondents for their time, and thankful for comments on a previous draft of the survey questionnaire from David Teira, Corinna Peters, Jan Sprenger, James Hampton, Christopher Boyce and Christian Krekel.

Author Contributions

Conceptualization: Alexander Krauss, Matteo Colombo.

Data curation: Alexander Krauss.

Formal analysis: Alexander Krauss.

Investigation: Alexander Krauss, Matteo Colombo.

Methodology: Alexander Krauss, Matteo Colombo.

Validation: Alexander Krauss, Matteo Colombo.

Visualization: Alexander Krauss.

Writing – original draft: Alexander Krauss, Matteo Colombo.

Writing – review & editing: Alexander Krauss, Matteo Colombo.

References

1. Douglas H. (2009). Science, policy, and the value-free ideal. Pittsburgh: University of Pittsburgh Press.
2. Kincaid H., Dupré J., & Wylie A. (Eds.). (2007). Value-Free Science: Ideals and Illusions? Oxford University Press.
3. Kitcher P. (2011).Science in a Democratic Society, New York: Prometheus Books.
4. Longino H. (1990).Science as Social Knowledge: Values and Objectivity in Scientific Inquiry, Princeton: Princeton University Press.
5. Merton R. K. (1973). The sociology of science: Theoretical and empirical investigations. University of Chicago press.

6. Oreskes N., & Conway E. M. (2011). Merchants of doubt: How a handful of scientists obscured the truth on issues from tobacco smoke to global warming. Bloomsbury Publishing USA.
7. Hornsey M. J., Harris E. A., Bain P. G., & Fielding K. S. (2016). Meta-analyses of the determinants and outcomes of belief in climate change. Nature Climate Change, 6(6), 622.
8. Blancke S., Tanghe K. B., &Braeckman J. (2018). Intuitions in science education and the public understanding of science. In Perspectives on science and culture, edited by Kris Rutten, Stefaan Blancke and Ronald Soetaert, 223–242. West Lafayette: Purdue.
9. Bromme R., & Goldman S. R. (2014). The public's bounded understanding of science. Educational Psychologist, 49(2), 59–69.
10. Clayton S., Devine-Wright P., Stern P. C., Whitmarsh L., Carrico A., Steg L., et al. (2015). Psychological research and global climate change. Nature Climate Change, 5(7), 640.
11. Colombo M., Bucher L., &Inbar Y. (2016). Explanatory judgment, moral offense and value-free science. Review of philosophy and psychology, 7(4), 743–763. https://doi.org/10.1007/s13164-015-0282-z PMID: 27917248
12. Corner A., Markowitz E., & Pidgeon N. (2014). Public engagement with climate change: the role of human values. Wiley Interdisciplinary Reviews: Climate Change, 5(3), 411–422.
13. Fernbach P. M., Rogers T., Fox C. R., & Sloman S. A. (2013). Political extremism is supported by an illusion of understanding. Psychological Science, 24(6), 939–946. https://doi.org/10.1177/0956797612464058 PMID: 23620547
14. Irwin A., & Wynne B. (Eds.). (2003). Misunderstanding science?: the public reconstruction of science and technology. Cambridge University Press.
15. Lewandowsky S., Ecker U. K., Seifert C. M., Schwarz N., & Cook J. (2012). Misinformation and its correction: Continued influence and successful debiasing. Psychological Science in the Public Interest, 13 (3), 106–131. https://doi.org/10.1177/1529100612451018 PMID: 26173286
16. Voelkel J. G., Brandt M. J., &Colombo M. (2018). I know that I know nothing: Can puncturing the illusion of explanatory depth overcome the relationship between attitudinal dissimilarity and prejudice?, Comprehensive Results in Social Psychology, 3:1, 56.
17. Lorenzoni I., & Pidgeon N. F. (2006). Public views on climate change: European and USA perspectives. Climatic change, 77(1–2), 73–95.
18. Whitmarsh L. (2009). What's in a name? Commonalities and differences in public understanding of "climate change" and "global warming". Public understanding of science, 18(4), 401–420.
19. Brulle R. J., Carmichael J., & Jenkins J. C. (2012). Shifting public opinion on climate change: an empirical assessment of factors influencing concern over climate change in the US, 2002–2010. Climatic change, 114(2), 169–188.
20. Whitmarsh L. (2011). Scepticism and uncertainty about climate change: Dimensions, determinants and change over time. Global environmental change, 21(2), 690–700.
21. Bisogni C. A., Jastran M., Seligson M. & Thompson A. (2012). How people interpret healthy eating: contributions of qualitative research. Journal of nutrition education and behavior, 44(4), 282–301. https://doi.org/10.1016/j.jneb.2011.11.009 PMID: 22732708
22. Jukola S. (2019). On the evidentiary standards for nutrition advice. Studies in History and Philosophy of Science Part C: Studies in History and Philosophy of Biological and Biomedical Sciences, 73, 1–9.
23. Shaw Hughner R., & Schultz Kleine S. (2008). Variations in lay health theories: Implications for consumer health care decision making. Qualitative health research, 18(12), 1687–1703. https://doi.org/10.1177/1049732308327354 PMID: 18955468
24. Ronteltap A., Sijtsema S. J., Dagevos H., & de Winter M. A. (2012). Construal levels of healthy eating. Exploring consumers' interpretation of health in the food context. Appetite, 59(2), 333–340. https://doi.org/10.1016/j.appet.2012.05.023 PMID: 22641147
25. Yarar N., & Orth U. R. (2018). Consumer lay theories on healthy nutrition: AQ methodology application in Germany. Appetite, 120, 145–157. https://doi.org/10.1016/j.appet.2017.08.026 PMID: 28851558
26. Blake C. E., Bisogni C. A., Sobal J., Devine C. M., & Jastran M. (2007). Classifying foods in contexts: how adults categorize foods for different eating settings. Appetite, 49(2), 500–510. https://doi.org/10.1016/j.appet.2007.03.009 PMID: 17512088
27. Falk L. W., Sobal J., Bisogni C. A., Connors M., & Devine C. M. (2001). Managing healthy eating: definitions, classifications, and strategies. Health education & behavior, 28(4), 425–439.
28. Roman S., Sánchez-Siles L. M., & Siegrist M. (2017). The importance of food naturalness for consumers: Results of a systematic review. Trends in Food Science & Technology, 67, 44–57.

29. Croll J. K., Neumark-Sztainer D., & Story M. (2001). Healthy eating: what does it mean to adolescents? Journal of nutrition education, 33(4), 193–198. https://doi.org/10.1016/s1499-4046(06)60031-6 PMID: 11953240

30. Paquette M. C. (2005). Perceptions of healthy eating: state of knowledge and research gaps. Canadian Journal of Public Health/Revue Canadienne de Sante'e Publique, S15–S19.

31. Bolitho F. H., Carr S. C., & Fletcher R. B. (2007). Public thinking about poverty: Why it matters and how to measure it. International Journal of Non profit and Voluntary Sector Marketing, 12(1), 13–22.

32. Panadero S., &Vazquez J. J. (2008). Perceived causes of poverty in developing nations: Causes of Third World Poverty Questionnaire in Spanish-speaking samples. Social Behavior and Personality: an international journal, 36(4), 571–576.

33. Will J. A. (1993). The dimensions of poverty: Public perceptions of the deserving poor. Social Science Research, 22(3), 312–332.

34. European Commission. (2010). Poverty and Social Exclusion. Brussels: European Commission, Eurobarometer. 2010, URL: https://ec.europa.eu/commfrontoffice/publicopinion/archives/ebs/ebs_321_en.pdf.

35. Astin J. A. (1998). Why patients use alternative medicine: results of a national study. Jama, 279(19), 1548–1553. https://doi.org/10.1001/jama.279.19.1548 PMID: 9605899

36. Colgan S., Faasse K., Martin L. R., Stephens M. H., Grey A., & Petrie K. J. (2015). Perceptions of generic medication in the general population, doctors and pharmacists: a systematic review. BMJ open, 5(12), e008915. https://doi.org/10.1136/bmjopen-2015-008915 PMID: 26671954

37. Furnham A., & Bhagrath R. (1993). A comparison of health beliefs and behaviours of clients of orthodox and complementary medicine. British Journal of Clinical Psychology. 32:237–46. https://doi.org/10.1111/j.2044-8260.1993.tb01051.x PMID: 8318944

38. Parsons S., Starling B., Mullan-Jensen C., Tham S. G., Warner K., &Wever K. (2015). What the public knows and wants to know about medicines research and development: a survey of the general public in six European countries. BMJ open, 5(4), e006420. https://doi.org/10.1136/bmjopen-2014-006420 PMID: 25854965

39. Isacson D., & Bingefors K. (2002). Attitudes towards drugs—a survey in the general population. Pharmacy world and science, 24(3), 104–110. https://doi.org/10.1023/a:1016127320051 PMID: 12136742

40. Vermeire E., Hearnshaw H., Van Royen P., & Denekens J. (2001). Patient adherence to treatment: three decades of research. A comprehensive review. Journal of clinical pharmacy and therapeutics, 26 (5), 331–342. https://doi.org/10.1046/j.1365-2710.2001.00363.x PMID: 11679023

41. Krauss Alexander. 2018. Why all randomised controlled trials produce biased results. Annals of Medicine, 50:4, 312–322, https://doi.org/10.1080/07853890.2018.1453233 PMID: 29616838

42. Dupré J. (2007). Fact and value. In Value free science: ideal or illusion?, eds. Harold Kincaid, John Dupré, Alison Wylie, 21–41. Oxford: Oxford University Press.

43. UNFCC. United Nations Framework Convention on Climate Change. (Article 1, para. 2). https://unfccc.int/resource/ccsites/zimbab/conven/text/art01.htm (Retrieved Jan 2020).

44. IPCC. Intergovernmetnal Panel on Climate Change. Climate Change. https://www.ipcc-data.org/guidelines/pages/glossary/glossary_c.html (Retrieved Jan 2020).

45. World Health Organisation. Nutritition. https://www.who.int/health-topics/nutrition (Retrieved Jan 2020).

46. World Bank. 1990. World Development Report 1990: Poverty. New York: Oxford University Press.

47. World Bank. 2001. World Development Report 2000/2001: Attacking Poverty. New York: Oxford University Press.

48. Cochrane. Glossary. London UK, https://community.cochrane.org/glossary (Retrieved Jan 2020).

49. Colquhoun D. 1998. Binding, gating, affinity and efficacy. The interpretation of structure–activity relationships for agonists and of the effects of mutating receptors. British Journal of Pharmacology. 125 (5), 923–948.

50. McCredie M. & Morey L. (2019). Who are the Turkers? A characterization of MTurk workers using the personality assessment inventory. Assessment, 26(5) 759–766. https://doi.org/10.1177/1073191118760709 PMID: 29490470

51. Levay K.,Freese J., & Druckman J. (2016). The Demographic and Political Composition of Mechanical Turk Samples. SAGE Open, 1–17.

52. Walters K, Christakis DA, Wright DR (2018). Are Mechanical Turk worker samples representative of health status and health behaviors in the U.S.? PLoS ONE 13(6): e0198835. https://doi.org/10.1371/journal.pone.0198835 PMID: 29879207

53. Freeden M., Sargent L.T. and Stears M (2013). The Oxford Handbook of Political Ideology. Oxford: Oxford University Press.

54. Maynard J. L. (2013). A Map of the Field of Ideological Analysis. Journal of Political Ideologies, 18(3), 299–327.
55. Kroh M. (2007). Measuring left–right political orientation: The choice of response format. Public Opinion Quarterly, 71(2), 204–220.
56. Noel A. and Therien J. (2008), Left and Right in Global Politics. Cambridge: CUP.
57. Rozenblit L., & Keil F. (2002). The misunderstood limits of folk science: An illusion of explanatory depth. Cognitive science, 26(5), 521–562. https://doi.org/10.1207/s15516709cog2605_1 PMID: 21442007
58. Alicke M. D. & Govorun O. (2005). The better-than-average effect. In Alicke M. D., Dunning D., & Krueger J. (Eds.), The self in social judgment (pp. 85–106). New York: Psychology Press.
59. Roberts D. (2013). Thick Concepts. Philosophy Compass, 8(8): 677–88.
60. Allum N., Sturgis P., Tabourazi D., & Brunton-Smith I. (2008). Science knowledge and attitudes across cultures: A meta-analysis. Public understanding of science, 17(1), 35–54.
61. Sturgis P., &Allum N. (2004). Science in society: re-evaluating the deficit model of public attitudes. Public understanding of science, 13(1), 55–74.
62. Elliott K. C. (2017). A tapestry of values: An introduction to values in science. Oxford University Press.
